# Damage Characteristics and Life Prediction of Desert Sand Concrete Under the Combined Effect of Continuous Axial Compressive Loading and Semi-Immersion in Mixed Salt

**DOI:** 10.3390/ma19143026

**Published:** 2026-07-14

**Authors:** Yuan Tian, Lei Gong, Ling Luo, Yongjun Qin, Xiaozhe Wang

**Affiliations:** 1 School of Civil Engineering and Architecture, Xinjiang University, Urumqi 830047, China; 2 Xinjiang Academy of Transportation Sciences Co., Ltd., Urumqi 830000, China; 3 Xinjiang Key Laboratory of Highway Engineering Technology in Arid Desert Region, Urumqi 830047, China; 4 Jiefubeng Expressway Branch, Anhui Transportation Holding Group Co., Ltd., Bengbu 233500, China

**Keywords:** desert sand concrete, semi-immersion, continuous axial load, mixed salt attack, nonlinear wiener process, life prediction

## Abstract

To evaluate the durability of desert sand concrete (DSC) in a semi-buried saline-alkali soil environment, this study examined DSC with a 30% desert sand replacement rate, using ordinary concrete (OC) as a control. A 180-day mixed salt attack test was conducted under continuous axial compressive loads (0, 30% *f_c_*, and 50% *f_c_*) and semi-immersion in a mixed salt solution (5% NaCl + 5% Na_2_SO_4_). Macroscopic and microscopic tests were conducted to reveal the damage evolution patterns of DSC, and a life prediction model was established using a nonlinear Wiener process. The results indicate that after 180 days of semi-immersion, under identical exposure media and load levels, DSC exhibited overall better durability retention compared with OC. A 30% *f_c_* load helped reduce pore connectivity in the DSC, suppressing the development of harmful and highly harmful pores and delaying performance degradation, whereas a 50% *f_c_* load promoted microcrack propagation and pore connectivity, accelerating degradation. Under mixed salt semi-immersion conditions, the “wick effect” caused distinct regional damage in DSC; compared with the corresponding soaking section, the adsorption section showed 3.84–5.17% lower *K_n_* values for compressive strength and a 25.57–42.52% higher proportion of harmful and highly harmful pores. The developed nonlinear Wiener model can reasonably characterize the relative degradation process of DSC under different exposure conditions, and the predicted trends are in good agreement with the results of macro- and micro-scale analyses.

## 1. Introduction

With ongoing infrastructure development, natural river sand resources are becoming increasingly scarce, and the search for suitable fine-aggregate substitutes has become a key topic in concrete materials research [1,2,3]. Desert sand is abundant in arid and semi-arid regions worldwide. Using it to partially replace river sand in the production of desert sand concrete (DSC) not only reduces concrete production costs but also mitigates the ecological damage caused by river sand extraction, yielding notable economic and environmental benefits. Consequently, research on the preparation, mechanical properties, and durability of desert sand concrete has garnered widespread attention from both the academic and engineering communities in recent years.

Existing research indicates that the appropriate incorporation of desert sand can improve the mechanical and durability properties of concrete. When the replacement rate is controlled within the range of 20% to 40%, the fine particles in desert sand can act as micro-fillers, improving the fine aggregate gradation and filling the initial pores and defects in the interfacial transition zone (ITZ), thereby enhancing the density of the concrete matrix [4,5,6]. At the same time, some studies suggest that desert sand may contain small amounts of reactive mineral components that, under certain conditions, participate in hydration reactions, improve the structure of the ITZ, and enhance the bonding performance between the paste and aggregates [7,8]. However, current research on desert sand concrete (DSC) has largely focused on conventional curing conditions or on a single aggressive factor; there remains a lack of systematic understanding of the long-term durability evolution and damage mechanisms of DSC in complex service environments characterized by the coupling of multiple factors.

Arid and semi-arid regions are not only rich in desert sand resources but also have widely distributed saline-alkali soils [9]. In such regions, evaporation is intense while leaching is minimal; soluble salts such as SO_4_^2−^ and Cl^−^ in the soil and shallow groundwater easily migrate with water and accumulate, posing a serious threat to the durability of local concrete infrastructure [10,11,12]. Among these, semi-buried structures such as bridge piles, retaining walls, and culverts typically operate with their lower portions embedded in saline soils or saline groundwater, while their upper portions are exposed to the atmosphere, resulting in particularly complex salt-attack exposure. Such structures are in prolonged contact with saline soil and saline groundwater. Salts migrate upward through capillary water along the internal pores of the concrete and continuously accumulate in the adsorption section above the water level. Consequently, the degree of deterioration in the adsorption section is typically greater than in the soaking section, resulting in distinct non-uniform damage patterns within the structure [13,14,15,16,17]. For DSC, the fine-grained nature of desert sand may further influence salt migration and the evolution of pore structure, such that the damage behavior of DSC in a semi-immersed salt-attack environment may differ from that of ordinary concrete.

In addition to salt attack, actual concrete members are subjected to their own weight and external loads over the long term during service. These loading conditions alter the internal pore and crack structures of the concrete, thereby affecting the transport pathways and rates of aggressive ions. Previous studies have shown that appropriate compressive stress can inhibit the transport of aggressive ions by compacting pores, whereas higher compressive stress may promote the initiation and propagation of microcracks, thereby accelerating the deterioration process [18,19,20]. However, most existing studies on the coupling of loading and salt attack have been conducted under full-immersion conditions, and research on the localized deterioration patterns in the adsorption and soaking sections of concrete under semi-immersed conditions remains insufficient. Therefore, it is necessary to conduct a systematic study of the evolution of DSC durability under the combined action of continuous axial compressive loading and mixed salt semi-immersion to elucidate the intrinsic relationships among load levels, salt migration, and localized damage.

Based on durability assessments, accurately predicting the life of concrete structures in aggressive environments is key to ensuring their durability and structural safety. Due to its independent increment property and the availability of an analytical solution for the first-passage time, the Wiener process can effectively describe the stochastic evolution of material performance degradation and has been widely applied to structural life prediction and reliability analysis [21,22,23]. Among these, the classical linear Wiener process is widely used due to its simplicity; however, its assumption of a constant drift rate has difficulty capturing the nonlinear acceleration in degradation rates observed in concrete during the later stages of exposure, which is caused by the accumulation of expansive products and the propagation of microcracks. To overcome this limitation, researchers have extended the Wiener process nonlinearly by introducing a time-scale transformation function *Λ*(*t*) *= t^q^* [24], with the parameter q characterizing the time-varying nature of the degradation rate (q > 1 indicates accelerated degradation, while q < 1 indicates decelerated degradation). This method retains the mathematical tractability of the Wiener process while effectively capturing the phased acceleration behavior of the degradation process. However, the applicability of this model in characterizing DSC degradation processes and predicting life under the coupled action of loading and mixed salt semi-immersion remains to be further verified.

Based on the above considerations, this study simulates the typical service environment of semi-buried concrete members in saline-alkali soil regions. It focuses on DSC with a 30% desert sand replacement rate and uses ordinary concrete (OC) without desert sand, prepared with the same cementitious system, as a control. The study systematically conducts clean water and mixed salt semi-immersion tests at different axial compressive stress levels. By combining macroscopic performance monitoring with microscopic testing, this study reveals the regional damage characteristics and degradation mechanisms of DSC under the coupled effects of loading and mixed salt semi-immersion. Building on these findings, a DSC life prediction model is developed based on a nonlinear Wiener process to quantitatively characterize relative durability degradation patterns, thereby providing a reference for long-term durability assessment and engineering applications of DSC in saline-alkali soil environments.

## 2. Materials and Methods

### 2.1. Raw Materials and Mix Proportions

#### 2.1.1. Cementitious Materials

The cementitious materials consisted of Tianshan P·O 52.5R ordinary Portland cement and highly reactive silica fume; their chemical composition is shown in Table 1. A polycarboxylate-based high-performance water-reducing agent with a water-reduction rate of 25% was used, and tap water was used for mixing.

#### 2.1.2. Aggregate

**Figure 1:**
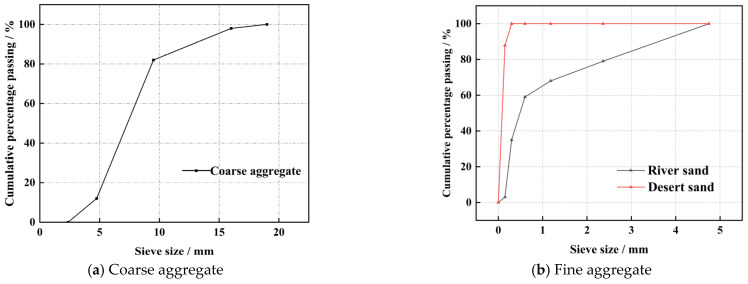
Particle size distribution curves of aggregates.

**Figure 2:**
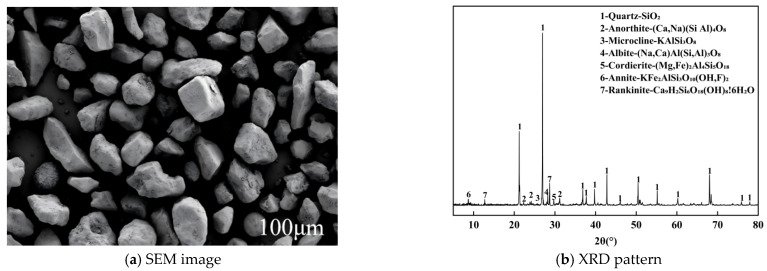
Desert sand: (**a**) SEM image; (**b**) XRD pattern.

**Figure 3:**
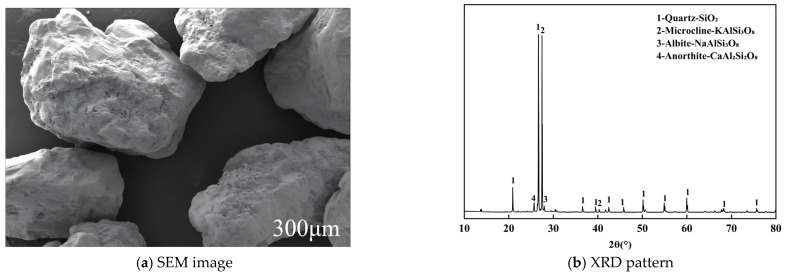
River sand: (**a**) SEM image; (**b**) XRD pattern.

Coarse aggregate consisted of natural river pebbles from the Xinjiang region, with a particle size range of 5–20 mm in a continuous gradation. The apparent density is 2740 kg/m^3^, the bulk density is 1530 kg/m^3^, the water absorption rate is 0.8%, the crushing index is 3.8%, and the content of elongated and flaky particles is 2.8%. The fine aggregate consisted of natural river sand and Taklamakan Desert sand. The river sand has a fineness modulus of 2.404, a particle size range of 0–5 mm, an apparent density of 2630 kg/m^3^, and a bulk density of 1610 kg/m^3^. The desert sand was sourced from the Taklamakan Desert. It has a fineness modulus of 0.122, an average particle size of 0.118 mm, an apparent density of 2790 kg/m^3^, and a bulk density of 1334 kg/m^3^. The extremely low fineness modulus indicates that desert sand contains a high proportion of fine particles, which may increase the specific surface area and water demand of the mixture and affect workability. To reduce this adverse effect, a polycarboxylate water-reducing agent was used to adjust the workability of the mixture. The particle size distribution curves for each aggregate are shown in Figure 1. The chemical composition of the desert sand is shown in Table 2. The SEM image and XRD pattern of desert sand are shown in Figure 2, whereas those of river sand are shown in Figure 3. As shown in Figure 2, the desert sand particles are relatively fine, with irregular surface morphology and predominantly subangular shapes. The XRD results indicate that the mineral composition of the desert sand is dominated by quartz, followed by feldspar, with small amounts of other minerals. These fine and irregular particles may improve particle packing in the concrete matrix and contribute to a micro-filling effect. In addition, the small amount of aluminosilicate minerals in desert sand may have potential reactivity. As shown in Figure 3, the river sand particles have a relatively larger particle size, irregular morphology, and rough surface texture. The XRD results indicate that river sand is mainly composed of quartz, with feldspar minerals such as microcline, albite, and anorthite also detected. Overall, both desert sand and river sand are mainly composed of silicate minerals, but they differ clearly in particle size and surface morphology. These differences may further affect the particle packing state, interfacial transition zone structure, and subsequent durability performance of DSC.

#### 2.1.3. Mix Design

The DSC mix design for this experiment was based on the research team’s previous findings, which showed that the mechanical properties of DSC first increased and then decreased with increasing desert sand replacement level. Among the investigated replacement levels, the 30% desert sand replacement rate exhibited relatively superior mechanical performance; therefore, this replacement rate was selected in this study as a representative optimized replacement level within the previously investigated material system [25]. Meanwhile, ordinary concrete (OC) without desert sand, prepared with the same cementitious material system, served as the control group. The detailed mix designs for each concrete group are shown in Table 3.

### 2.2. Specimen Preparation and Grouping

#### 2.2.1. Specimen Preparation

Both DSC and OC specimens were prepared as prisms measuring 100 mm × 100 mm × 400 mm. After molding, the specimens were cured in a standard curing chamber for 28 days. Upon completion of curing, the initial relative dynamic modulus of elasticity, initial mass, and 28-day reference compressive strength (*f_c_*) were determined for both DSC and OC specimens. Subsequently, the continuous axial compressive load levels were determined using the 28-day reference compressive strength (*f_c_*) for each group, and the specimens were loaded to the corresponding target stress prior to semi-immersion exposure testing. During the test, the relative dynamic modulus of elasticity and mass of the DSC specimens were measured every 30 days, and compressive strength tests were conducted at corresponding ages of 30, 60, 90, 120, 150, and 180 days; the OC control group underwent relative dynamic modulus of elasticity, mass, and compressive strength tests only after 180 days of exposure to evaluate the effect of desert sand incorporation on the durability performance of concrete. Therefore, all subsequent comparisons between the DSC and OC groups are based on the test results at 180 days of age.

#### 2.2.2. Grouping of Test Specimens

This experiment employed two exposure media and three levels of continuous axial compressive loading. Based on field surveys of saline-alkali soils in Xinjiang [26] and relevant studies on load–salt-attack coupling [27], clean water was selected as the control medium, and a mixed salt solution (5% NaCl + 5% Na_2_SO_4_) was used as the accelerated mixed salt attack medium, with both 5% NaCl and 5% Na_2_SO_4_ expressed as mass fractions. The levels of continuous axial compressive load were set at 0%, 30%, and 50% of the 28-day compressive strength (*f_c_*) of the test specimens. Specifically, 0% represents the unloaded control; 30% *f_c_* simulates low-stress conditions during the elastic compression stage, where appropriate continuous compressive stress helps consolidate the matrix; and 50% *f_c_* simulates high-stress conditions during the plastic compression stage, increasing the likelihood of microcrack initiation and propagation.

**Figure 4:**
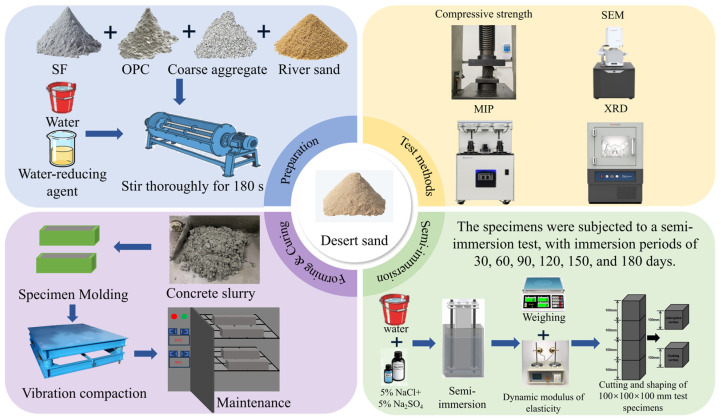
Test flow diagram.

The test groupings are shown in Table 4, where D and O denote desert sand concrete and ordinary concrete, respectively; CW and MS denote clean water and mixed salt solution, respectively; and 0, 30, and 50 denote the three continuous axial compressive load levels of 0%, 30%, and 50% *f_c_*, respectively. The semi-immersion period was 180 days, and the test procedure is shown in Figure 4.

### 2.3. Loading and Exposure Methods

#### 2.3.1. Continuous Axial Loading

A custom-built continuous axial compression loading device developed by the research team was used to apply axial compressive loads to the DSC and OC specimens. After 28 days of standard curing, the specimens were loaded to the target stress level at a rate of 0.5 MPa/s using a universal testing machine, and the load was locked in place by tightening the nuts. The loading device incorporated disc springs between the bolts and the steel plates to minimize load loss due to concrete creep and steel relaxation. During the test, the bolt pretension was verified every 7 days using a torque wrench. The load retention was evaluated by checking the bolt torque and converting it to the corresponding axial force according to the pre-established torque-load calibration relationship. Monitoring results showed that the load loss rate did not exceed 5% throughout the test period, indicating that the loading device exhibited good load-holding stability. Concrete creep was not separately quantified in this study, and its influence was mainly reflected in the measured load loss during the loading period.

#### 2.3.2. Semi-Immersion Test

Before the semi-immersion test, the liquid level was marked at the midpoint of the test specimen’s height. During the test, the specimens were placed in the exposure tanks, and the liquid level was maintained at this mark so that the lower half of each specimen was immersed and the upper half was exposed to air. In this paper, the region above the liquid surface—which is jointly influenced by capillary adsorption, evaporation, crystallization, and salt accumulation—is referred to as the adsorption section, while the region below the liquid surface that is continuously immersed in the solution is referred to as the soaking section. The test environment temperature was (20 ± 2) °C, and the relative humidity was (60 ± 5)%. During the test, the solution was replenished at regular intervals to maintain a constant liquid level, and the mixed salt solution was replaced every 30 days to maintain solution concentration.

### 2.4. Durability Testing

Durability testing was conducted in accordance with the “Standard for Test Methods of Long-term Performance and Durability of Concrete” (GB/T 50082—2024) [28]. Prior to testing, loose debris and salt crystals were removed from the test specimens’ surfaces, and any visible surface water was wiped away to minimize the impact of surface contaminants and free water on the test results. For each group at each test age, three parallel specimens were tested, and the reported values were the arithmetic means of the three measurements. The error bars in the figures in Section 3.2, Section 3.3 and Section 3.4 represent ±1 standard deviation. Due to the limited number of parallel specimens, formal statistical significance testing was not performed in this study. Therefore, the comparisons among different groups should mainly be interpreted as relative variation trends rather than statistically verified significant differences.

#### 2.4.1. Test Method for Relative Dynamic Modulus of Elasticity

The transverse fundamental frequency of the test specimens was measured using a dynamic modulus of elasticity tester, and the relative dynamic modulus of elasticity was calculated according to Equation (1):

(1) $$E_{dr}=\frac{E_{dn}}{E_{d0}}$$

In the equation, *E_dr_* represents the relative dynamic modulus of elasticity; *E_dn_* represents the dynamic modulus of elasticity of the specimen after *n* days of semi-immersion; and *E_d_*_0_ represents the initial dynamic modulus of elasticity of the specimen.

#### 2.4.2. Test Method for Mass Loss Rate

The mass loss rate quantifies the change in a test specimen’s mass during exposure due to water absorption, product deposition, dissolution, and surface spalling. It is an important indicator for evaluating the extent of macroscopic deterioration in concrete. Its calculation formula is given in Equation (2):

(2) $$W_{n}=\frac{G_{0}-G_{n}}{G_{0}}\times 100\%$$

In the equation, *W_n_* represents the mass loss rate (%); *G*_0_ represents the initial mass of the specimen (g); and *G_n_* represents the mass of the specimen after *n* days of semi-immersion (g). A negative value for *W_n_* indicates an increase in the specimen’s mass, while a positive value indicates a loss of mass.

#### 2.4.3. Test Method for Cube Compressive Strength

Under semi-immersion conditions, there are differences in water migration, salt accumulation, and evaporation crystallization between the adsorption and soaking sections; the compressive strength of each section can be used to evaluate the extent of mechanical property degradation in different areas. Prior to the compressive strength test, the prismatic specimens were cut along the marked positions at the liquid surface to obtain test blocks from the adsorption section above the liquid surface and the soaking section below it, respectively, which were then machined into 100 mm × 100 mm × 100 mm cubic specimens. The compressive strength of the cubes from both sections was tested separately. Three specimens were selected for each age, and the results were averaged. The salt-attack resistance coefficient for compressive strength was then calculated using Equation (3):

(3) $$K_{n}=\frac{f_{y}}{f_{q}}$$

In the equation, *K_n_* is the salt-attack resistance coefficient for compressive strength; *f_y_* is the compressive strength (MPa) of the corresponding region of the specimen after *n* days of semi-immersion in a mixed salt solution; *f_q_* is the compressive strength (MPa) of the corresponding region of the specimen after *n* days of semi-immersion in clean water.

### 2.5. Microscopic Testing

After the 180-day exposure test, samples were collected from the adsorption and soaking sections of the DSC specimens, respectively. All samples were broken into small pieces of approximately 5–10 mm, immersed in anhydrous ethanol to halt hydration, and then vacuum-dried to constant weight for later use. Because this study focuses on revealing the regional damage mechanism of DSC under the coupled action of continuous axial compressive loading and mixed salt semi-immersion, the microscopic tests were mainly performed on DSC specimens.

#### 2.5.1. SEM Test Method

After gold sputtering, the test samples were examined using an SU8600 field-emission scanning electron microscope (Hitachi, Tokyo, Japan) to observe the microstructures of the different test groups.

#### 2.5.2. XRD Test Method

After the test samples were ground into powder, their phase compositions were analyzed using a D8 X-ray diffractometer (Bruker, Karlsruhe, Germany). The tests were conducted using Cu Kα radiation at an operating voltage of 40 kV and a current of 40 mA, with a scanning rate of 5°/min and a 2θ scanning range of 5° to 80°.

#### 2.5.3. MIP Test Method

The pore structure parameters, including porosity, pore size, and pore size distribution, of samples from different test groups were quantitatively analyzed using an AutoPore V 9600 fully automated mercury intrusion porosimeter (Micromeritics, Norcross, GA, USA).

## 3. Results and Discussion

### 3.1. Appearance

**Figure 5:**
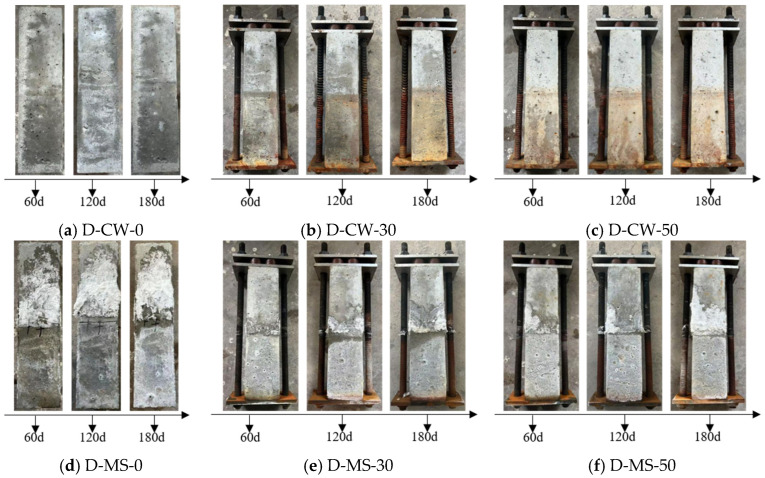
Surface morphology of DSC after different semi-immersion durations under different exposure media and load levels.

Figure 5 shows the surface morphology of the DSC specimens after different semi-immersion durations. The specimens in the clean water group exhibited relatively mild surface degradation. The surfaces of the D-CW-0, D-CW-30, and D-CW-50 specimens remained generally intact, with no obvious through cracks or large-scale surface spalling observed. Under different load levels, the D-CW-30 specimens exhibited relatively good surface integrity, while the D-CW-50 specimens showed slight spalling and fine cracks at some corners.

The specimens in the mixed salt group exhibited relatively pronounced apparent deterioration, with clear morphological differences between the adsorption and soaking sections. In the adsorption section, as the semi-immersion duration increased, white salt crystals gradually extended upward from the liquid surface, and the number of surface microcracks increased. This phenomenon is primarily related to the “wick effect” under semi-immersion conditions [29], whereby the mixed salt solution enters the interior of the concrete via capillary action and migrates upward along the pores to the adsorption section above the liquid surface. As water evaporates from the surface of the adsorption section, the pore solution gradually concentrates and becomes supersaturated. When the local pore solution reaches a supersaturated state, NaCl gradually crystallizes and precipitates, while Na_2_SO_4_ may crystallize and precipitate in forms such as mirabilite [30], generating crystallization pressure. Simultaneously, the concentrated SO_4_^2−^ and Cl^−^ ions react with cement hydration products to form salt-attack reaction products, such as ettringite and Friedel’s salt [31]. As salt crystals and reaction products continue to accumulate in the pores and microcracks, their crystallization pressure and volumetric expansion constantly exert pressure on the pore walls, promoting the initiation, propagation, and eventual interconnection of microcracks, ultimately leading to an increase in surface cracks and spalling of the surface layer in the adsorption section. The load level also influences the apparent degree of deterioration of the specimens. At a semi-immersion duration of 180 days, the D-MS-30 specimen exhibited relatively low salt crystallization coverage on the surface of the adsorption section and mild apparent deterioration. This preliminarily indicates that continuous axial compression at 30% *f_c_* can reduce the pore connectivity of DSC through compaction, thereby inhibiting the capillary migration and salt accumulation of the mixed salt solution and alleviating deterioration in the adsorption section [32]. In the soaking section, the lower half of the specimen remained continuously submerged, resulting in minimal water evaporation. Consequently, the pore solution was less likely to reach supersaturation, and physical crystallization was not pronounced. Additionally, the “wick effect” caused some salts to migrate upward with capillary water into the adsorption section, leading to relatively lower salt accumulation in the soaking section. Therefore, the apparent degree of deterioration in the soaking section was generally milder than that in the adsorption section [33].

### 3.2. Relative Dynamic Modulus of Elasticity

**Figure 6:**
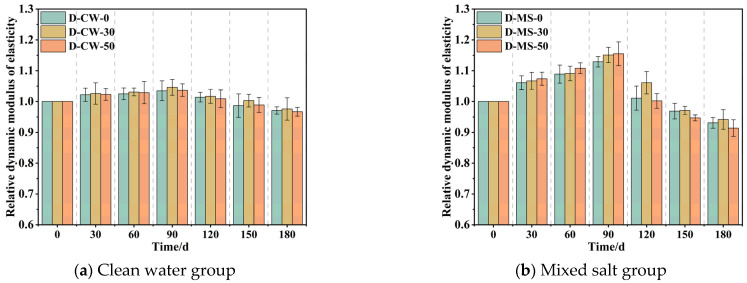
Relative dynamic modulus of elasticity of DSC during semi-immersion under different exposure media and load levels.

Figure 6 shows the evolution of the relative dynamic modulus of elasticity of DSC during semi-immersion exposure. All DSC specimens in the clean water group exhibited an initial increase followed by a decrease, with the peak occurring at 90 days; among them, D-CW-30 had the highest peak value of 1.046. During the early stages of exposure, water penetrated into the concrete matrix via capillary action, promoting continuous cement hydration. The resulting hydration products, such as calcium silicate hydrate (C-S-H) gel, filled internal pores, increasing the matrix density and causing the relative dynamic modulus of elasticity to rise [34]. After 90 days, the relative dynamic modulus of elasticity began to decrease, primarily due to two factors: first, the dissolution of some hydration products, which reduced the solid phase and increased porosity [35]; second, humidity differences between the adsorption section and the soaking section under semi-immersion conditions led to non-uniform deformation and additional internal stresses within the specimens, which in turn accelerated microcrack propagation [36]. The combined effect of these two factors reduced matrix density, thereby decreasing the relative dynamic modulus of elasticity. At 180 days, the relative dynamic moduli of elasticity of D-CW-0, D-CW-30, and D-CW-50 had decreased by 2.9%, 2.4%, and 3.3%, respectively, compared with their initial values, indicating a relatively low overall degree of damage.

The mixed salt groups also exhibited a trend of initially rising and then declining, with all groups reaching their peaks at 90 days; moreover, the decline following the peak was more pronounced than that of the clean water group. During the early stage of mixed salt attack, salt crystallization and reaction products such as gypsum and ettringite filled internal pores and microcracks, making the matrix more compact and increasing its relative dynamic modulus of elasticity [37]; in the later stages, the continuous accumulation of these crystalline and reaction products generated crystallization pressure and volumetric expansion that gradually exceeded the ultimate tensile strength of the DSC pore walls, leading to the continuous development and interconnection of microcracks within the matrix and an accelerated decline in the relative dynamic modulus of elasticity [38]. At 180 days, the relative dynamic moduli of elasticity of D-MS-0, D-MS-30, and D-MS-50 were 0.931, 0.942, and 0.914, respectively, with D-MS-30 exhibiting the best retention capacity and D-MS-50 the lowest. This indicates that a 30% *f_c_* axial compressive load can, to some extent, slow down the degradation of the dynamic modulus of elasticity and suppress damage accumulation, whereas a 50% *f_c_* axial compressive load promotes microcrack development, thereby accelerating the degradation of the dynamic modulus of elasticity [39].

### 3.3. Mass Loss Rate

**Figure 7:**
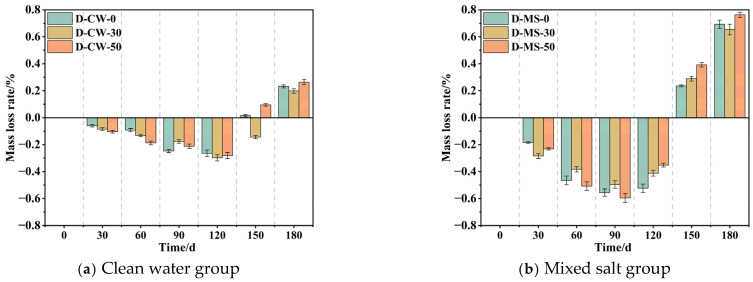
Mass loss rate of DSC during semi-immersion under different exposure media and load levels.

Figure 7 shows the evolution of the mass loss rate of DSC during semi-immersion exposure. The mass of the DSC specimens in the clean water group first increased, then decreased. Between 0 and 120 days, the mass loss rates for all groups were negative, indicating that the specimens were in a mass gain phase; at 120 days, the maximum mass gains of D-CW-0, D-CW-30, and D-CW-50 were 0.264%, 0.298%, and 0.282%, respectively. After 120 days, the specimen mass gradually decreased; at 180 days, the mass loss rates for D-CW-0, D-CW-30, and D-CW-50 were 0.232%, 0.198%, and 0.264%, respectively. This indicates that capillary water absorption and continuous hydration dominated the early stage of the semi-immersion test, with water entering the specimens’ interiors and promoting hydration; the resulting hydration products filled the pores, causing the specimens’ mass to increase. In the later stage, some of the hydration products dissolved, and the humidity gradient may have induced microcrack formation within the specimens, which then propagated toward the surface, causing slight spalling and a decrease in the specimens’ mass. Overall, the mass change in the clean water group was relatively small, indicating that the effect of semi-immersion in clean water alone on DSC mass stability was limited.

The mixed salt group specimens also exhibited an initial increase followed by a decrease, but the peak mass gain occurred earlier than in the clean water group, at 90 days. At 90 days, the maximum mass gains of D-MS-0, D-MS-30, and D-MS-50 were 0.556%, 0.496%, and 0.596%, respectively, primarily due to salt crystallization and the deposition of reaction products filling the pores during the early stages of mixed salt attack. As the semi-immersion period was extended, these products accumulated, and their crystallization pressure and volumetric expansion gradually caused internal damage. When cracks extended to the surface and corners, spalling occurred, leading to accelerated mass loss [40]. At 180 days, the mass loss rates for D-MS-0, D-MS-30, and D-MS-50 were 0.693%, 0.654%, and 0.763%, respectively, with D-MS-30 showing the lowest rate and D-MS-50 the highest. This indicates that appropriate continuous axial compressive loading helps maintain the specimen’s mass stability, whereas higher loads promote microcrack propagation and the transport of aggressive ions, thereby exacerbating mass loss under mixed salt attack.

### 3.4. Compressive Strength

**Figure 8:**
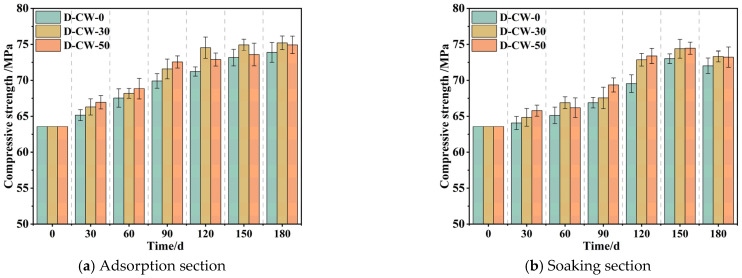
Compressive strength of the adsorption and soaking sections of DSC during clean water semi-immersion at different load levels.

**Figure 9:**
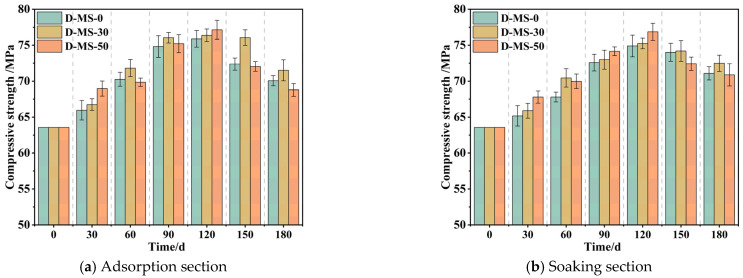
Compressive strength of the adsorption and soaking sections of DSC during mixed salt semi-immersion at different load levels.

Figure 8 and Figure 9 show the evolution of compressive strength in the adsorption and soaking sections of DSC under clean water and mixed salt semi-immersion conditions, respectively. The compressive strength values reported in this section are the directly measured strengths of the corresponding adsorption and soaking sections. In the clean water group, the compressive strength in the DSC adsorption section continued to increase throughout the test period, whereas that in the soaking section decreased slightly after 150 days. Since the adsorption section is located above the liquid surface, capillary water migration can supply moisture for subsequent hydration, leading to continuous strength gain [41]; in contrast, the soaking section remains saturated for an extended period, resulting in slight dissolution of late-stage hydration products and the development of internal microcracks, which causes a slight decrease in strength. At 180 days, the compressive strengths of the adsorption sections for D-CW-0, D-CW-30, and D-CW-50 were 73.89, 75.21, and 74.93 MPa, respectively, while those of the soaking sections were 72.02, 73.31, and 73.22 MPa, respectively. Based on the 180-day results, the compressive strength of the 30% *f_c_* group was higher than those of the 50% *f_c_* and unloaded groups.

The compressive strength in the DSC adsorption and soaking sections for the mixed salt group showed an initial increase, followed by a decrease, with peak values at 120 days. At 120 days, the compressive strengths of the adsorption sections for D-MS-0, D-MS-30, and D-MS-50 were 75.88, 76.38, and 77.14 MPa, respectively, while those of the soaking sections were 74.89, 75.24, and 76.86 MPa, respectively. Before 120 days, salt crystallization and the deposition of reaction products partly filled pores and microcracks, increasing the density of the matrix and leading to a short-term increase in strength; after 120 days, salt crystallization and expansive reaction products continued to accumulate, and the dominant mechanism gradually shifted from the initial filling effect to an expansive cracking effect, causing pores and microcracks to develop further and resulting in a decrease in compressive strength. At 180 days, D-MS-30 exhibited the highest compressive strength in both the adsorption and soaking sections, at 71.51 MPa and 72.48 MPa, respectively; D-MS-50 exhibited the lowest compressive strength in both sections, at 68.78 MPa and 70.87 MPa, respectively. This indicates that continuous axial compression at 30% *f_c_* can, to some extent, delay the strength loss caused by mixed salt attack, whereas continuous axial compression at 50% *f_c_* exacerbates damage development in the later stages.

The salt-attack resistance coefficient *K_n_* was calculated using Equation (3). After 180 days, the *K_n_* values for the adsorption sections of D-MS-0, D-MS-30, and D-MS-50 were 0.948, 0.951, and 0.918, respectively, while those for the soaking sections were 0.987, 0.989, and 0.968, respectively. Using the *K_n_* values of the soaking sections within the same group as a reference, the *K_n_* values of the adsorption sections for D-MS-0, D-MS-30, and D-MS-50 decreased by 3.95%, 3.84%, and 5.17%, respectively. This indicates that DSC exhibits pronounced localized damage under mixed salt semi-immersion conditions. This is mainly because the adsorption section is located above the liquid surface, where salt is more likely to accumulate, and local pores and microcracks are subjected to stronger crystallization and expansion effects; in contrast, the soaking section is constantly immersed in the solution, where concentrated salt crystallization is weaker, resulting in relatively milder degradation [42,43]. Together, these results indicate that, under mixed salt semi-immersion, the adsorption section was the primary deterioration zone; meanwhile, the beneficial effect of 30% *f_c_* and the adverse effect of 50% *f_c_* were consistently reflected in the relative dynamic modulus of elasticity, mass loss rate, and compressive strength results.

### 3.5. Comparison of Durability Metrics for OC and DSC

To further evaluate the effect of desert sand incorporation on the durability of concrete, the key durability indicators of the OC and DSC groups at 180 days were compared, and the results are shown in Table 5. The directly measured compressive strengths of the adsorption and soaking sections are also included in Table 5 as the original strength data used to calculate the salt-attack resistance coefficient. As shown in Table 5, under the same exposure medium and load levels, the DSC groups generally exhibited better durability retention than the corresponding OC groups. Specifically, under mixed salt semi-immersion conditions, compared with the corresponding OC groups, DSC showed a 4.78–6.65% higher relative dynamic modulus of elasticity, an 8.15–10.47% lower mass loss rate, and 3.49–6.52% and 4.32–5.68% higher *K_n_* values in the adsorption and soaking sections, respectively. These improvements may be mainly attributed to the filling effect of an appropriate amount of fine desert sand particles, which optimizes the concrete pore structure and enhances the compactness of the ITZ, thereby slowing the transport of aggressive ions and improving the resistance of DSC to degradation in semi-immersed salt-attack environments [44].

### 3.6. Microstructure

#### 3.6.1. SEM

**Figure 10:**
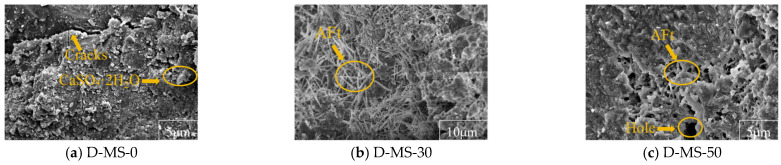
SEM images of the DSC adsorption section after 180 d of mixed salt semi-immersion under different load levels.

**Figure 11:**
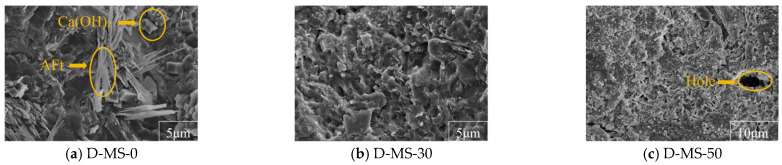
SEM images of the DSC soaking section after 180 d of mixed salt semi-immersion under different load levels.

SEM observations were performed on samples from the adsorption and soaking sections of the D-MS-0, D-MS-30, and D-MS-50 specimens after 180 days of semi-immersion; the results are shown in Figure 10 and Figure 11.

As shown in Figure 10, clear differences were observed in the distribution of reaction products and in the extent of matrix damage among the three adsorption-section samples. In the D-MS-0 adsorption section (Figure 10a), a large number of plate-like crystal deposits were observed, and continuous cracks appeared on the matrix surface, indicating that crystal deposition and expansion had caused pronounced damage to the matrix. In the D-MS-30 adsorption section (Figure 10b), a large number of needle-rod-shaped crystals were observed, with no obvious through-cracks in the matrix; the structure was generally denser than that of D-MS-0, indicating that an appropriate continuous axial compressive load improved the matrix density to some extent. In the D-MS-50 adsorption section (Figure 10c), plate-like and needle-rod-shaped crystals coexisted, accompanied by distinct voids and cracks, indicating the most pronounced microstructural degradation. This suggests that a continuous compressive stress of 50% *f_c_* promotes the propagation of microcracks within the matrix and the formation of pore connectivity, providing additional pathways for the ingress of SO_4_^2−^ and Cl^−^. In the late stages of mixed salt attack, salt crystals and reaction products continue to accumulate within pores and cracks, and their expansion further accelerates matrix degradation.

As shown in Figure 11, reaction-product accumulation in the soaking section was less pronounced than that in the corresponding adsorption section; the cracks and pores appeared smaller, and the matrix was generally more compact. In the D-MS-0 soaking section (Figure 11a), a small number of needle-like and plate-like crystals were observed, and the crystals had not yet caused obvious cracking. In the D-MS-30 soaking section (Figure 11b), less reaction-product accumulation was observed, and the matrix was relatively dense. In the D-MS-50 soaking section (Figure 11c), the voids were relatively prominent, but the extent of damage was still less severe than in its corresponding adsorption section. Overall, the soaking sections were subjected to weaker evaporation and crystallization effects, resulting in less crystal deposition, smaller pores and cracks that were not obviously interconnected, and relatively mild microstructural degradation.

#### 3.6.2. XRD

**Figure 12:**
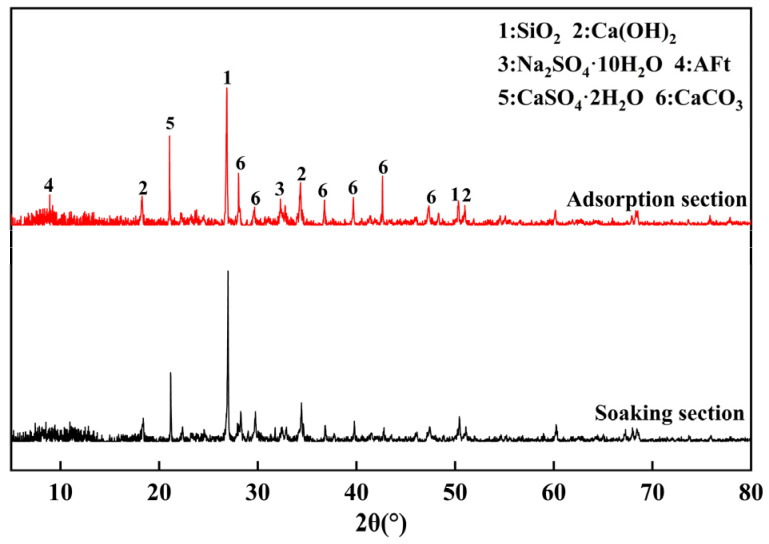
XRD patterns of the adsorption and soaking sections of D-MS-50 after 180 d of mixed salt semi-immersion.

Given that the D-MS-50 group exhibited the most pronounced deterioration in macroscopic properties after 180 days of semi-immersion, it was selected as the representative severely deteriorated group for XRD analysis. Powder samples from the adsorption and soaking sections of the D-MS-50 specimens were collected to identify the primary crystalline products generated during mixed salt attack. The results are shown in Figure 12. As shown in Figure 12, characteristic diffraction peaks of ettringite (AFt) and gypsum were detected in both the adsorption section and soaking section samples. Based on the needle-like and plate-like crystal morphologies observed in SEM, the combined SEM and XRD results suggest that reaction products such as ettringite and gypsum were present in both the adsorption and soaking sections of the specimens. These products initially fill pores and microcracks, but their continued accumulation in later stages leads to squeezing- and expansion-induced cracking, consistent with the crystal deposition, voids, and cracks observed in SEM. No Friedel’s salt diffraction peaks were detected in this XRD analysis. This may be related to the fact that, in the later stages of mixed salt attack, SO_4_^2−^ promotes the decomposition of Friedel’s salt and its conversion to ettringite [45], or to the fact that the amount of Friedel’s salt was below the XRD detection limit.

Furthermore, the intensity of the sodium sulfate decahydrate (Na_2_SO_4_·10H_2_O) diffraction peak was higher in samples from the adsorption section than in those from the soaking section, indicating that under semi-immersion conditions, salt is more likely to accumulate and crystallize in the region above the liquid surface. It should be noted that this XRD result reflects sulfate-related crystalline products retained in the solid powder samples after exposure and sample preparation, rather than indicating that Na_2_SO_4_·10H_2_O directly formed in the bulk solution. The phase transition of sodium sulfate crystals is accompanied by considerable volume change [46], and the resulting crystallization and expansion stress can further accelerate the development of pores and cracks, thereby exacerbating microstructural damage in the adsorption section.

In addition to chloride and sulfate attack, the Na^+^ introduced by NaCl and Na_2_SO_4_ may also affect the alkalinity and ionic strength of the pore solution, especially in the adsorption section where evaporation promotes salt accumulation. However, the independent effect of Na^+^ or alkali-related reactions was not specifically evaluated in this study, and no direct evidence of alkali-related reaction products was obtained from the present SEM and XRD results. Therefore, based on the available experimental results, the deterioration mechanism is mainly discussed in terms of chloride attack, sulfate attack, and salt crystallization, while the possible alkali-related effect is acknowledged as a potential contributing factor. Moreover, due to experimental limitations, quantitative phase analysis was not performed in this study; therefore, the XRD results were mainly used for qualitative identification of reaction products.

### 3.7. Pore Structure

**Figure 13:**
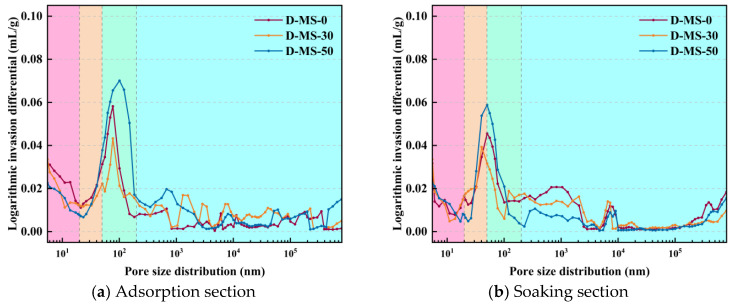
Pore size distribution of DSC adsorption and soaking sections after 180 d of mixed salt semi-immersion under different load levels.

**Figure 14:**
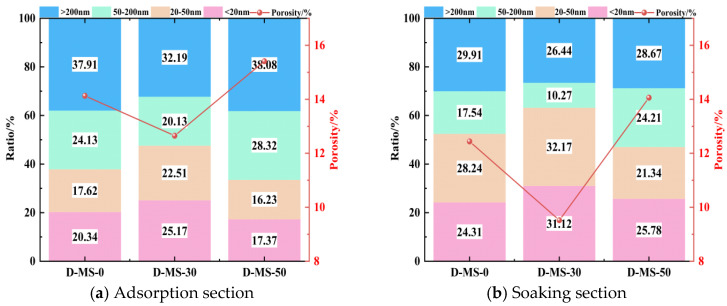
Porosity and pore proportions of DSC adsorption and soaking sections after 180 d of mixed salt semi-immersion under different load levels.

MIP tests were conducted on samples from the adsorption and soaking sections of the D-MS-0, D-MS-30, and D-MS-50 specimens after 180 days of semi-immersion. Although pore harmfulness classifications vary in the literature, this study adopts the pore size classification criterion in Ref. [47] for comparative analysis. Based on this criterion, the pores were categorized into harmless pores (d < 20 nm), slightly harmful pores (20 nm ≤ d < 50 nm), harmful pores (50 nm ≤ d < 200 nm), and highly harmful pores (d ≥ 200 nm). The resulting pore size distributions, porosity, and pore proportions are shown in Figure 13 and Figure 14.

The most probable pore diameter is the pore diameter corresponding to the peak in the pore size distribution curve and is an important parameter for characterizing pore structure [48]. As shown in Figure 13, the most probable pore diameters in the adsorption sections of D-MS-0, D-MS-30, and D-MS-50 were 77.09, 77.14, and 100.76 nm, respectively, all of which fell within the harmful pore range; in the soaking section, the most probable pore diameter of D-MS-30 was 40.32 nm, which fell within the slightly harmful pore range, while those of D-MS-0 and D-MS-50 were 50.38 and 50.42 nm, respectively, both falling within the harmful pore range. Overall, the most probable pore diameters in the adsorption sections of all groups were greater than those in the corresponding soaking sections, indicating that the coarsening of the pore structure was more pronounced in the adsorption sections.

As shown in Figure 14, the porosity of the adsorption sections for D-MS-0, D-MS-30, and D-MS-50 was 14.13%, 12.65%, and 15.41%, respectively, while that of the soaking sections was 12.44%, 9.52%, and 14.06%, respectively; the proportions of harmful and highly harmful pores in the adsorption sections were 62.04%, 52.32%, and 66.40%, respectively, while those in the soaking sections were 47.45%, 36.71%, and 52.88%, respectively. Compared with the soaking section within the same group, the proportions of harmful and highly harmful pores in the adsorption sections of D-MS-0, D-MS-30, and D-MS-50 were 30.75%, 42.52%, and 25.57% higher, respectively. This indicates that the adsorption sections are more strongly affected by capillary adsorption, evaporation crystallization, and salt accumulation, resulting in a greater degree of pore structure degradation than in the soaking sections.

Under different load levels, the D-MS-30 specimen exhibited the lowest porosity and the lowest proportions of harmful and highly harmful pores within the same region, while the D-MS-50 specimen exhibited the highest values. Compared with D-MS-0, the porosity of D-MS-30 decreased from 14.13% to 12.65% in the adsorption section and from 12.44% to 9.52% in the soaking section, corresponding to relative decreases of 10.47% and 23.47%, respectively. In contrast, the porosity of D-MS-50 increased to 15.41% and 14.06% in the adsorption and soaking sections, corresponding to relative increases of 9.06% and 13.02%, respectively. These quantitative MIP results indicate that an axial compressive load of 30% *f_c_* can densify the matrix, close some of the original voids, and reduce transport channels, whereas an axial compressive load of 50% *f_c_* may induce microcracks, promote void connectivity, and increase ion transport paths. Therefore, the load-dependent changes in porosity and pore size distribution provide quantitative support for the interpretation that appropriate continuous axial compressive loading is beneficial for improving the pore structure of DSC, whereas higher continuous axial compression exacerbates pore structure degradation [39]. This interpretation is also consistent with previous studies showing that appropriate compressive loading may reduce pore connectivity by compacting the pore structure, whereas higher sustained loading can promote connected microcracks and accelerate the transport of aggressive ions [18,19,20].

## 4. DSC Life Prediction Based on Nonlinear Wiener Theory

### 4.1. Damage Assessment Indicators

Based on the theory of concrete damage mechanics, the relative dynamic modulus of elasticity is selected as an indicator of durability damage. The relative dynamic modulus of elasticity loss rate is defined as the damage variable *X*(*t*) [49], expressed as follows:

(4) $$X\left(t\right)=1-E_{dr}\left(t\right)$$

where *X*(*t*) is the damage variable of the DSC at time t; *E_dr_*(*t*) is the relative dynamic modulus of elasticity of the DSC at time t. According to the provisions of the “Standard for Test Methods of Long-term Performance and Durability of Concrete” (GB/T 50082—2024) [28], durability failure is determined when the relative dynamic modulus of elasticity of the concrete specimen decreases to 60% (i.e., the loss rate reaches 40%); therefore, the failure threshold *D_f_* is set to 0.4 in this study. This threshold was used as a comparative durability criterion for different test conditions rather than as an absolute structural failure criterion for field members. Since an increase in the relative dynamic modulus of elasticity may occur in the early exposure stages, *X*(*t*) may take on negative values initially. This study treats this as a random fluctuation during the degradation process and uses the first time at which the damage variable reaches the failure threshold as the life criterion.

### 4.2. Construction of a Nonlinear Wiener Process Model

The traditional linear Wiener process assumes a constant drift rate, making it difficult to capture the nonlinear, accelerated degradation characteristics exhibited by DSC in the late stages of exposure, driven by the accumulation of expansive products and the propagation of microcracks. To address this, this study introduces a time-scale transformation function Λ(*t*) *= t^q^* [24] to construct a nonlinear Wiener model:

(5) $$X(t)=\mu \Lambda (t)+\sigma B\left(\Lambda (t)\right)=\mu t^{q}+\sigma B\left(t^{q}\right)$$

In the equation: *μ* is the drift parameter, which characterizes the degradation rate; *σ* is the diffusion parameter, which characterizes the degree of fluctuation; *B*(*⋅*) represents standard Brownian motion; *q* is the time-scale parameter, which characterizes the nonlinear variation in the degradation rate: when *q* > 1, it indicates accelerated degradation; when *q* < 1, it indicates decelerated degradation; and when *q* = 1, the model reduces to a linear Wiener process.

The predicted failure time T under DSC accelerated exposure conditions is defined as the time at which the damage variable first reaches or exceeds the failure threshold *D_f_*, that is:

(6) $$T=inf\{t\geq 0:X(t)\geq D_{f}\}$$

According to the Fokker–Planck equation [50], the probability density function of the damage variable is given by

(7) $$p(x,t)=\frac{1}{\sigma \sqrt{2\pi t^{q}}}\left\{\exp\left(-\frac{{\left(x-\mu t^{q}\right)}^{2}}{2\sigma^{2}t^{q}}\right)-\exp\left(\frac{2\mu D_{f}}{\sigma^{2}}\right)\exp\left(-\frac{{\left(x-2D_{f}-\mu t^{q}\right)}^{2}}{2\sigma^{2}t^{q}}\right)\right\}$$

This yields the reliability function *R*(*t*)

(8) $$R(t)=\Phi \left(\frac{D_{f}-\mu t^{q}}{\sigma \sqrt{t^{q}}}\right)-\exp\left(\frac{2\mu D_{f}}{\sigma^{2}}\right)\Phi \left(\frac{-D_{f}-\mu t^{q}}{\sigma \sqrt{t^{q}}}\right)$$

In the equation, Φ(x) is the cumulative distribution function of the standard normal distribution.

By differentiating Equation (8) and taking into account the Jacobian term resulting from the time-scale transformation, we obtain the probability density function of T:

(9) $$f(t)=\frac{D_{f}qt^{q-1}}{\sigma \sqrt{2\pi {\left(t^{q}\right)}^{3}}}\exp\left(-\frac{{\left(D_{f}-\mu t^{q}\right)}^{2}}{2\sigma^{2}t^{q}}\right)$$

### 4.3. Model Parameter Estimation

Model parameters were estimated using maximum likelihood estimation (MLE). All model fitting, parameter estimation, statistical analyses, bootstrap resampling, life prediction calculations, and figure generation were performed using MATLAB (R2024b). Let the observed data be *X*_0_:*n* = {*x*_0_, *x*_1_, *x*_2_*,* …, *x_n_*}, and the corresponding observation times be *t*_0_:*n* = {*t*_0_, *t*_1_, *t*_2_, …, *t_n_*}. The transformed time increments and damage increments are defined as:

(10) $$\Delta \tau_{i}=t_{i}^{q}-t_{i-1}^{q},\;\Delta x_{i}=x_{i}-x_{i-1},\;i=1,2,\ldots ,n$$

Based on the independent increment property of the Wiener process, the damage increment Δ*x_i_* follows a normal distribution, Δ*_xi_*~*N* (*μ*Δ*_τi_*, *σ^2^*Δ*_τi_*). The likelihood function is defined as:

(11) $$L(\mu ,\sigma^{2}|q)=\prod_{i=1}^{n}\frac{1}{\sqrt{2\pi \sigma^{2}\Delta \tau_{i}}}\exp\left[-\frac{{\left(\Delta x_{i}-\mu \Delta \tau_{i}\right)}^{2}}{2\sigma^{2}\Delta \tau_{i}}\right]$$

Taking the partial derivatives of Equation (11) with respect to *μ* and *σ^2^*, respectively, and setting them equal to zero yields the maximum likelihood estimate for a given *q*:

(12) $$\hat{\mu }(q)=\frac{\sum_{i=1}^{n}\Delta x_{i}}{\sum_{i=1}^{n}\Delta \tau_{i}}$$

(13) $${\hat{\sigma }}^{2}(q)=\frac{1}{n}\sum_{i=1}^{n}\frac{{\left(\Delta x_{i}-\hat{\mu }(q)\Delta \tau_{i}\right)}^{2}}{\Delta \tau_{i}}$$

Since *q* appears in a nonlinear form in Δ*_τi_*, the Profile Likelihood method is used for estimation, and the estimated value can be expressed as:

(14) $$\hat{q}=\underset{q}{\arg\max}\;\ln L\left(\hat{\mu }(q),{\hat{\sigma }}^{2}(q),q\right)$$

By substituting the DSC degradation data under different exposure conditions into Equations (12)–(14), the model parameters *μ*, *σ*^2^, and *q* for the specimens under different exposure conditions were calculated, as shown in Table 6.

### 4.4. Model Verification and Uncertainty Analysis

To examine whether the normal increment assumption of the Wiener process was reasonable for the experimental data, the normality of the DSC damage increments under various operating conditions was verified. The damage increments for each group were standardized as *z_i_*:

(15) $$z_{i}=\frac{\Delta x_{i}-\hat{\mu }\Delta \tau_{i}}{\hat{\sigma }\sqrt{\Delta \tau_{i}}}$$

**Figure 15:**
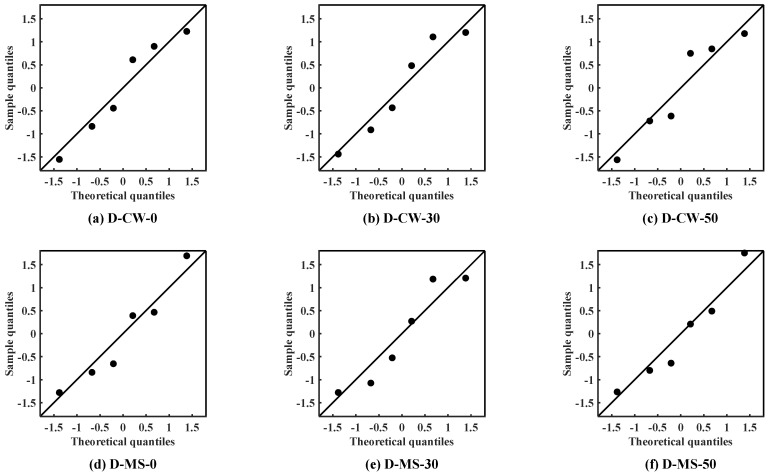
Q–Q plots of standardized increments for the nonlinear Wiener model.

The Q–Q plots for each group are shown in Figure 15; the standardized increments are distributed approximately along the 45° reference line. The results of the K–S test are presented in Table 7; the *p*-values for all groups are greater than 0.05, indicating that, given the current sample size, the hypothesis that the standardized increments follow a normal distribution is not rejected. Furthermore, 1000 bootstrap resampling iterations were performed on the damage increment sequences to re-estimate the model parameters μ, σ^2^, and q and to construct 95% prediction intervals for the degradation process. The measured damage values at all test ages fell within the prediction intervals, indicating that the developed model can reasonably characterize the degradation trends under the present experimental conditions. However, no independent external validation dataset was available in this study. Therefore, the predicted life should be regarded as an estimated result under the present accelerated exposure test conditions rather than a fully independently verified long-term prediction. Future studies will further validate the proposed model using independent long-term exposure data and field monitoring data.

### 4.5. DSC Life Prediction

**Figure 16:**
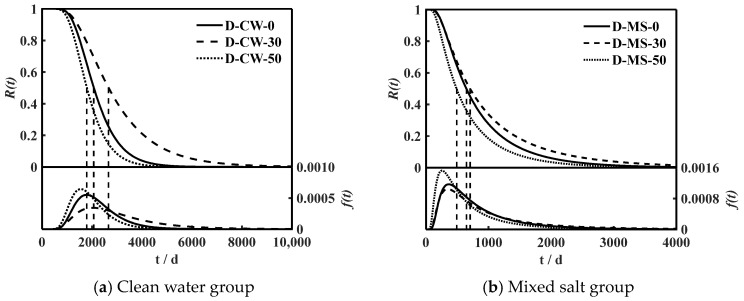
Reliability and probability density functions of DSC life predicted by the nonlinear Wiener model.

Figure 16 shows the reliability functions and probability density functions for the predicted life of DSC. As shown in Figure 16, the reliability of DSC generally decreases with increasing semi-immersion duration. Under clean water semi-immersion conditions, the *q*-values for D-CW-0, D-CW-30, and D-CW-50 were 1.043, 0.998, and 1.052, respectively, all of which are close to 1. This indicates that the degradation process in the clean water group is approximately linear, with a relatively gradual decline in reliability. This is because, under clean water semi-immersion conditions, the initial hydration reaction continues, and the hydration products gradually densify the internal structure; as the exposure duration increases, the humidity difference between the adsorption section and the soaking section causes an uneven distribution of internal stress, and at the same time, some hydration products dissolve, leading to an increase in the damage variable and a gradual decline in reliability.

In contrast, the *q*-values for D-MS-0, D-MS-30, and D-MS-50 were 1.120, 1.111, and 1.144, respectively, all greater than 1.1. This indicates that DSC degradation under mixed salt semi-immersion conditions exhibits a distinct nonlinear acceleration, with a relatively steep decline in the reliability curve. This is because, under semi-immersion in a mixed salt solution, reaction products fill the internal pores in the early stages, maintaining reliability at a relatively high level; as the semi-immersion duration increases, reaction products continue to accumulate, and damage caused by their expansive effects gradually becomes dominant, accelerating the damage accumulation rate and causing reliability to decline sharply [49]. Among these groups, D-MS-50 exhibits the highest *q*-value and the largest drift parameter *μ*, indicating that damage accumulation is fastest in the later exposure stages under the coupled action of a 50% *f_c_* load and mixed salt semi-immersion. In summary, the time-scale parameter *q* of the nonlinear Wiener model can effectively characterize the evolution of DSC degradation rates, and its predicted trends are generally consistent with the results of macroscopic performance degradation and microscopic damage analyses.

Taking into account the requirements for durability design and safety assessment, this study uses the median life [51], i.e., the time corresponding to *R*(*t*) = 0.5, as the predicted failure time under DSC accelerated exposure conditions. As shown in Figure 16, the predicted lives for D-CW-0, D-CW-30, D-CW-50, D-MS-0, D-MS-30, and D-MS-50 were 2072 d, 2664 d, 1794 d, 644 d, 702 d, and 487 d, respectively. Compared with D-MS-0, the predicted life of D-MS-30 increased by approximately 9.0%, while that of D-MS-50 decreased by approximately 24.4%. These results indicate that appropriate sustained compressive loading can delay durability degradation, and DSC has potential for engineering applications in semi-buried concrete structures in saline-alkali desert regions. The predicted life of the clean water group was generally higher than that of the mixed salt group, indicating that mixed salt attack markedly shortened the predicted life of DSC. Under both clean water and mixed salt semi-immersion conditions, the 30% *f_c_* group showed a relatively longer predicted life, whereas the 50% *f_c_* group showed a shorter predicted life. This suggests that appropriate sustained compressive loading can improve the relative durability performance of DSC, whereas excessive stress accelerates degradation. This is consistent with the aforementioned experimental findings that a 30% *f_c_* load delays degradation, while a 50% *f_c_* load accelerates damage. From an engineering perspective, the use of 30% desert sand improved the durability retention of concrete under the tested mixed salt semi-immersion environment. Compared with the corresponding OC groups at 180 days, DSC showed a 4.78–6.65% higher relative dynamic modulus of elasticity, an 8.15–10.47% lower mass loss rate, and 3.49–6.52% and 4.32–5.68% higher *K_n_* values in the adsorption and soaking sections, respectively. These improvements suggest that DSC has potential for application in semi-buried concrete members in saline-alkali desert regions, such as bridge piles, retaining walls, and culverts.

## 5. Conclusions

In this study, the damage evolution of DSC was investigated through a 180-day coupled exposure test involving continuous axial loading and semi-immersion in a mixed salt solution. By combining macro- and micro-scale testing with a nonlinear Wiener process, a life prediction model was established. The main conclusions are as follows:

(1) Under mixed salt semi-immersion conditions and the same load levels, DSC exhibited better durability retention than the corresponding OC group at 180 days. Compared with the corresponding OC groups, DSC showed a 4.78–6.65% higher relative dynamic modulus of elasticity, an 8.15–10.47% lower mass loss rate, and 3.49–6.52% and 4.32–5.68% higher *K_n_* values in the adsorption and soaking sections, respectively. This indicates that the incorporation of 30% desert sand helps improve the durability retention of concrete in a semi-immersed salt environment.
(2) Continuous axial compressive loading has a clear effect on the durability of DSC. Under a 30% *f_c_* load, the DSC exhibited the smallest decrease in relative dynamic modulus of elasticity, the lowest mass loss rate, and the lowest proportions of harmful and highly harmful pores, indicating that appropriate continuous axial compressive loading helps reduce pore connectivity and delay the transport of aggressive ions. Compared with D-MS-0, the porosity of D-MS-30 decreased by 10.47% in the adsorption section and by 23.47% in the soaking section. In contrast, under a 50% *f_c_* load, microcracks and connected pores increased, and deterioration was accelerated. Compared with D-MS-0, the porosity of D-MS-50 increased by 9.06% in the adsorption section and by 13.02% in the soaking section.
(3) Under mixed salt semi-immersion conditions, the “wick effect” caused salt to accumulate more readily in the DSC adsorption section, resulting in a higher degree of degradation in the adsorption section compared with the soaking section. At 180 days, compared with the corresponding soaking sections, the adsorption sections showed 3.84–5.17% lower *K_n_* values for compressive strength and a 25.57–42.52% higher proportion of harmful and highly harmful pores. This indicates that the adsorption section is the key deterioration region for semi-buried concrete members exposed to saline environments.
(4) A DSC life prediction model based on a nonlinear Wiener process was developed using the relative loss rate of the dynamic modulus of elasticity as the damage variable. The *q*-values for the clean water group ranged from 0.998 to 1.052, approaching 1, indicating an approximately linear degradation process; the *q*-values for the mixed salt group were all greater than 1.1, indicating nonlinear, accelerated degradation in the later stages. The predicted median lives of D-CW-0, D-CW-30, D-CW-50, D-MS-0, D-MS-30, and D-MS-50 were 2072 d, 2664 d, 1794 d, 644 d, 702 d, and 487 d, respectively. Compared with D-MS-0, the predicted life of D-MS-30 increased by approximately 9.0%, while that of D-MS-50 decreased by approximately 24.4%. The predicted trends were in good agreement with the results of macroscopic performance degradation and microscopic damage analyses, and the model can provide a reference for evaluating the relative service life of DSC under different operating conditions.

This study has several limitations. First, the experiments were conducted using a single mix design with a 30% replacement rate of desert sand; therefore, the degradation patterns under different replacement rates and salt concentrations require further verification. Second, the OC control specimens were mainly evaluated at 180 days, and direct SEM, XRD, and MIP comparisons between DSC and OC were not conducted. Therefore, the microscopic deterioration differences between DSC and OC require further investigation in future work. Third, although mean values and standard deviations were reported for the main durability indicators, formal statistical significance testing was not performed because of the limited number of parallel specimens. Fourth, the surface deterioration shown in Figure 5 was mainly evaluated through visual observation, and quantitative crack-width measurements or surface-damage indices were not obtained. Fifth, the life predictions were extrapolated from 180-day accelerated exposure test data. While the results primarily reflect relative differences in degradation across operating conditions, the assessment of actual life in engineering applications still requires further refinement through longer-term testing and field exposure data.

## Figures and Tables

**Table 1:** Chemical composition of cementitious materials (%).

Composition	CaO	SiO_2_	Al_2_O_3_	Fe_2_O_3_	SO_3_	MgO	Na_2_O	K_2_O	TiO_2_	MnO
Cement	62.77	20.65	5.33	3.19	4.01	2.56	0.19	0.77	0.24	0.04
Silica fume	0.01	97.03	0.55	0.18	—	0.41	0.01	0.39	0.01	—

**Table 2:** Chemical composition of desert sand (%).

Composition	SiO_2_	Al_2_O_3_	CaO	Fe_2_O_3_	K_2_O	Na_2_O	MgO	TiO_2_	P_2_O_5_
Content	66.47	14.48	4.48	3.23	2.71	2.56	2.24	0.67	0.25

**Table 3:** Mix proportions of concrete (kg/m^3^).

Group	Water	Silica Fume	Cement	Desert Sand	River Sand	Pebble	Water Reducer
DSC	155.00	31.91	423.97	196.67	458.90	1165.46	6.78
OC	155.00	31.91	423.97	0	655.57	1165.46	6.78

**Table 4:** Specimen groups.

Concrete Type	Solution	0	30% *fc*	50% *fc*
DSC	Clean water	D-CW-0	D-CW-30	D-CW-50
DSC	Mixed salt solution	D-MS-0	D-MS-30	D-MS-50
OC	Clean water	O-CW-0	O-CW-30	O-CW-50
OC	Mixed salt solution	O-MS-0	O-MS-30	O-MS-50

**Table 5:** Durability indicators and corresponding compressive strength data of OC and DSC after 180 d semi-immersion exposure.

Specimen	Relative Dynamic Modulus of Elasticity	Mass Loss Rate/%	Compressive Strength of Adsorption Section/Mpa	Compressive Strength of Soaking Section/Mpa	*K*_n_ of Adsorption Section	*K*_n_ of Soaking Section
O-CW-0	0.937	0.306	71.92	70.12	—	—
D-CW-0	0.971	0.232	73.89	72.02	—	—
O-CW-30	0.955	0.212	73.38	71.78	—	—
D-CW-30	0.976	0.198	75.21	73.31	—	—
O-CW-50	0.941	0.271	72.41	71.52	—	—
D-CW-50	0.967	0.264	74.93	73.22	—	—
O-MS-0	0.885	0.774	64.01	65.84	0.890	0.939
D-MS-0	0.931	0.693	70.06	71.09	0.948	0.987
O-MS-30	0.899	0.712	67.36	68.04	0.918	0.948
D-MS-30	0.942	0.654	71.51	72.48	0.951	0.989
O-MS-50	0.857	0.834	64.23	65.51	0.887	0.916
D-MS-50	0.914	0.763	68.78	70.87	0.918	0.968

Note: “—” indicates that the clean water group is not included in the calculation of the salt-attack resistance coefficient *K_n_* for compressive strength.

**Table 6:** Key parameters of the nonlinear Wiener model.

Specimen	*q*	*μ*	*σ* ^2^
D-CW-0	1.043	1.292 × 10^−4^	8.367 × 10^−6^
D-CW-30	0.998	1.348 × 10^−4^	1.458 × 10^−5^
D-CW-50	1.052	1.399 × 10^−4^	8.965 × 10^−6^
D-MS-0	1.120	2.056 × 10^−4^	6.686 × 10^−5^
D-MS-30	1.111	1.807 × 10^−4^	7.829 × 10^−5^
D-MS-50	1.144	2.263 × 10^−4^	9.245 × 10^−5^

**Table 7:** K–S test results of the nonlinear Wiener model.

Specimen	D-CW-0	D-CW-30	D-CW-50	D-MS-0	D-MS-30	D-MS-50
*p*	0.850	0.934	0.673	0.796	0.892	0.812

## Data Availability

The original contributions presented in the study are included in the article, further inquiries can be directed to the corresponding author.
